# Introductory Address upon Southern Institutions, Publicly Delivered October 13, 1879

**Published:** 1879-12-20

**Authors:** T. S. Powell

**Affiliations:** Professor of Obstetrics in Southern Medical College


### Southern Medical Record.

EDITORS:

T. S. POWELL, M.D. W. T. GOLDSMITH, M.D. R. C. WORD, M.D.

*R. C. WORD, M.D., Managing Editor.*

☞All Communications and Letters on Business connected with the RECORD must be addressed to the Managing Editor.

VOL. IX. ATLANTA, GA., DECEMBER 20, 1879. NO. 12.

### ORIGINAL AND SELECTED ARTICLES.

### *INTRODUCTORY ADDRESS UPON SOUTHERN INSTITUTIONS.*

PUBLICLY DELIVERED OCTOBER 13, 1879, BY T. S. POWELL, M.D.,

Professor of Obstetrics in Southern Medical College.

*Ladies and Gentlemen:*

It was my desire to have one of my colleagues deliver the introductory address of the first course of lectures in the *Southern Medical College*, but as this duty has been pressed upon me, I have accepted it with grateful appreciation of the honor, and with a sincere wish to say something that will entertain this respected and intelligent audience.

In these latter days it is becoming fashionable to ignore the truth of the Bible, nevertheless I am inclined to select as the ground work of these remarks a passage from that Holy Book—a passage in which is comprehended a volume of logic that cannot be refuted—of ethics that cannot be perverted, and general premises that no man can remove from their Divinely established positions. The passage to which I refer is this: "Whatsoever a man soweth, that shall he reap." I have never yet known a man or woman to have the temerity to dispute the truth of this assertion, but as I do not design to deliver a lecture on morality, I quote this Biblical passage only in the application of its general truth, and to make it the basis of my subject proper, which I propose to be

SOUTHERN INSTITUTIONS.

In this wide-awake era in the Nineteenth Century, I doubt if there is an intelligent child among us of ten years of age, who is not more or less impressed with the idea that this is an age of inspiring progression—that transition from state to a still higher and better attainment in all things that add to the beauty, the comfort and the general well being of our race, is characteristic of the present state of human action. Onward is but the natural order of all created things. It is the watchword of Nature, and man echoes the cry in response to her cheering voice; but to man alone, inspired by the Infinite, is accorded the privilege of adding the word "upward," as he thinks, and plans, and earnestly acts for the benefit and happiness of his fellow-man, for the high prestige of his native land and the boundaries of his whole country. Progression was an incentive to the ancient races, as it is to us in this age. The sovereigns of Egypt were stimulated by its action when they erected monuments of such vast proportions and wonderful skill that the generations of more than two thousand years have looked with amazement upon these marvelous memorials of mechanical art and labor. The Greeks and the Romans, urged on by its ev r-active spirit, built magnificent temples, organized national institutions, gave to the world glorious examples of art, and investigations of science so far as was possible with the limited resources of their age.

No step made in progressive movement is ever lost to the world, and we of to-day are the beneficiaries of every improvement made in those periods of the mighty past. But as the utmost limit of perfection attainable at one period of the world's history is far behind that arrived at "by generations that follow," new improvements must supplant old ones; and antiquated inventions, theories, and modes of action give place to the more enlightened ones of advanced knowledge and ever-progressive industrial enterprise. While every improvement for the human race will either immediately or in the course of time be felt throughout the world, it is evident to every man that those progressive movements that will most materially benefit his own section or community should first have his favor and support. Not from a selfish motive or sectional prejudice, but from the higher consideration of developing the talent or industry that will benefit others who are nearest and dearest by the bond of association, and from the love of locality implanted in the heart of every true man. As the All-wise Supreme Being has linked all men together from the rivers to the ends of the earth, with the ever-throbbing, magnetic chain of human sympathy and fraternal interest—every movement, every recorded instance, and every institution for the benefit and elevation of the human race finds an instantaneous and sympathetic response in all the hearts of those who love their fellow men, so soon as the news is flashed along the wires from one continent to another, or from section to section of our common country. A good deed done in China, India or the South Sea Islands causes a thrill of joy in the soul of every good man and woman in North America. We hear of associations organized and institutions erected for promoting the comfort and interest or developing talent upon the soil of Europe, or in different sections of our own country, but these institutions, these organizations—aside from their local habitation—are cosmopolitan. They belong to the "wide, wide world;" their influence is for the whole race of man—Christian, Jew, or Gentile. As the rays of the full, unclouded moon penetrate every nook and corner of the forest and silently throw their lovely beams even under the leaves of the shrinking violet, so the light of every noble work, with its attendant ministration, reaches the most obscure depths where the human race is found. Can you suppose that the grandeur, the beauty, and the magnificence of St. Peter's, St. Paul's—the cathedrals at Milan and Strasburg—were designed for only the eye and the worship of the inhabitants in their respective cities? The lovely creations of Roman and Greek art, in painting or sculpture, were not merely for the artistic glory of Rome or Greece, but men and women of almost every nation have drank inspiration from their beauty, and modeled from their immortal forms and color.

The "Home, Sweet Home" of John Howard Payne is a stereotyped melody around the fireside in every land where the song has been sung, and the beautiful self-immolated life of his illustrious namesake is a precious legacy to the whole philanthropic world. In our own country the time-honored names of Yale and Harvard are not enshrined only in the memory of their beneficiaries among our Northern friends—but North, East, South and West have paid their tribute of eulogy to these halls of learning, and in every section of the Union there are found alumnæ of these institutions, who to the close of their lives will have vivid and grateful recollections of their loved and revered *Alma Mater*. The venerable William and Mary and Randolph-Macon Colleges, though erected upon Southern soil, open their fountains of knowledge to every one, irrespective of clime or nationality, who would drink of the Pierian spring, and the more modern institution, Washington and Lee College, has already a world-wide fame, wherever the names of these, our illustrious Chieftains, are known and honored. It is but natural that our own Southern institutions, whether national, scholastic, industrial or philanthropic, should be the more closely entwined about the Southern heart, as their interest, their prestige and their prosperity are more individually our own. We are the more direct recipients of their benefits; we feel the more sympathy in their character and utility; for the sake of our children and future generations, we more earnestly desire their perpetuity, and we feel a pride in their establishment in proportion to the reputation and the worth of the institutions themselves. Who can so fully estimate the worth of Vanderbilt University to the South, as we of its sunny land, and can Southern hearts cease to remember with grateful memorials the names of its noble and generous founders? The University of Georgia, Mercer University and Emory College have an honored memory throughout the Union, but to none can they be so dear as to the people of Georgia. During the last twelve years there has been a greater impetus given to the establishment of Southern institutions than perhaps the fifty preceding ones. The South is now awake to the importance of a movement in this direction that will keep pace with the progressive age, and of such a character as will presage the future of Southern greatness.

Within the last decade hundreds of factories, or other establishments representing varied industrial pursuits, have been erected upon our soil—educational interests are of a more diffusive and higher character than ever before in our section, and organizations of literature, art and science are now established in localities where a few years ago there was not one temple to do homage to these divinities of an enlightened progression. No man will deny that it is his *patriotic* duty to support and encourage every institution erected upon his native soil that will add to the honor and the prosperity of his section or entire country. The South, with its almost limitless possibilities in mind and industry; with its magnificent natural resources; its political character and talents; with its men and women of genius, and delightful climate, has in a pre-eminent degree all the agents from which to evolve the full grown statue of a country's national, educational, industrial and financial greatness. Her grand historic record is but the horoscope of her future—a horoscope star—bright with the prophetic greatness of the sunny South.

The city of Atlanta has many advantages to make it the Southern nucleus of medical science and exposition: Its climate all the year round; its varied and continually increasing population; its energy, wealth, enterprise, public libraries and medical talent all combine to offer advantages and facilities of a high character to the medical student, and enable him to pursue his studies with both pleasure and profit. We repeat that it is the patriotic duty of the State and the South to encourage and support an institution of this character that now offers itself to the favor of the public and the people of our city. There are unfortunately too many pessimists in our communities; too many who look upon all things as a misfortune or calamity—that cast an "evil eye" upon every worthy movement, and predict for it utter failure in the end. Though the optimist is the extreme antithesis of this character, it is much better to have its cheering tones to greet us; its visions of naught but prophetic good, and its inspiring action in every onward and upward movement. As regards medical schools and men, the pessimists look upon them all for the worst. They either have no need for the colleges and doctors, or the colleges are not all they should be, and the doctors quacks, humbugs, impostors, etc. Others take the medium ground, and say that our law and medical colleges are equal to our necessities, and serve our purpose well enough. This is the answer of indifference and mediocrity to intelligence and progress. They forget that the spirit of medical education, like that of all other genuine instruction, is "Let knowledge grow from more to more." No individual, no city or community, and no institution should accept mediocrity when excellence, and even eminence can be attained. In no progression and in no institution is this excellence more important and desirable than in medicine and medical colleges. Though much is yet to be achieved by the medical profession in this country, its graduates can compare favorably with those in law and other professions, and I doubt if there are not as many good doctors as there are lawyers, merchants, farmers, etc. Apropos, to quote from a pertinent speaker on this subject. He says: "Men learned in law and medicine are not popular with demagogues, but in their conceit and ignorance they seem to think that any man who can read, write and cipher is qualified to be a lawyer or doctor." This idea would lower the standard of medicine to the statue of every quack and impostor, and make such legal lights as shone in a certain circuit court of which I once heard. The Judge was not much learned in technicalities, knew but little Latin, much less Greek. The jury was composed of ordinary farmers. A smart and saucy lawyer by the name of Brown rose to defend a case. He spoke two hours in the highest possible style, soaring aloft, repeating Greek and bringing a shower of technical terms to the end of his tongue. The jury sat with open mouths, the Judge stared with amazement; Brown closed—the verdict of the jury was against him—motion for a new trial. In the morning Brown rose and bowed to the court: "May it please your Honor, I humbly rise this morning to move for a new trial. On yesterday I gave wings to my imagination and rose above the stars in a blaze of glory. I saw at the time that it was all Greek and Turkey tracks to you and the jury. This morning I feel humble, and I promise the court, if they will grant me a new trial, I will bring myself down to the comprehension of the court and jury." "Motion overruled," says the court, "and a fine of five dollars against Mr. Brown for contempt of court." "For what?" exclaimed Brown. "For insinuating that this court don't know Latin and Greek from turkey tracks."

It is my opinion that the achievements in medicine in this country will not suffer by comparison with those in Europe, when we take into consideration the superior antiquity and facilities of the profession in the latter country. An over-weaning self-confidence in any pursuit is detrimental to success, but just appreciation is not conceit, and modest modicum of boasting is better than self-abasement. The words of inspiring hope and encouragement should be the rule to develop the talent among the people and ensure success to every worthy enterprise. Every tone of this kind will be a mite to cast into the common treasury of agents that incite a man's confidence in his efforts and his cause. In elevating the standard of medical character and education, and giving them their legitimate rank and protection, the public welfare will gain rather than lose. The people of the State and the State itself should not be more indifferent to the character and capability of physicians than they are to that of teachers in their public schools.

No man should have and does have more of the spirit of progressive knowledge than the true physician, but to elevate the profession to the desired eminence he must have the sympathy and support of the public; its confidence in his efforts, and its respect for them. The object and the earnest desire of the founders of the Southern Medical College of Atlanta is to legitimately and thoroughly teach the science and the practice of medicine upon a broad, high and progressive basis. Its establishment was not designed from any motives of rivalry to other institutions, but in a spirit of emulation of laudable ambition, and upon the universally recognized principle of live and let live in every undertaking for the common good. Some may say, "We have too many doctors now, as we have too many lawyers." I will admit that the doctors and "Colonels" are equally divided, and that there are tricksters in medicine as well as in law, or trade; yet we do not forget the trite but famous reply of Daniel Webster: "There is always room at the top," and the design of this medical institution is to bring both professors and students to the highest round of the ladder. It is by pursuing this course in all its departments, mental, moral and medical, that we hope to make the institution eminently worthy of favor and patronage. We desire to begin at the beginning upon this immovable platform, knowing that in this as in all things, "Whatsoever a man soweth, that shall he reap." If the genuine seed of medical knowledge is sown here in good ground, the public will reap a harvest of practitioners that will be an honor to the institution and the profession, and a blessing to the human race. Besides, a new school of medicine here will not increase the number of students in the aggregate; it will only increase the number in Atlanta, and thus benefit the city.

A word to our students, and I will close. We cordially welcome you, young gentlemen, to our city, and to the initial exercises of the Southern Medical College. In the name of the Faculty, I present their kindest wishes for your success in attaining the best and highest knoweledge in the study of medicine. Next to the ministry of the Gospel, you have chosen the noblest and grandest of all professions, and it remains with you to become true and worthy members of the fraternity. A solid education, a close and patient investigation of your work, must be the basis upon which to build your future efficiency and reputation. The science of medicine is no more speculative theory: it is not a haphazard system or series of reckless experiments, but it is a grand collective science, composed of philosophical principles that are mathematically true. You must prove them, classify and master them before you are fitted for the practice of the medical art, and become a healer indeed of the ills that beset our physical humanity; and remember, you are not educating yourselves to be merely physicians—but accomplished gentlemen, men of strict honesty and integrity, men of conscientious scruples and unsullied honor, men of energetic and progressive enterprise in your profession, men who reverence God and his laws, men with kind, sympathetic hearts, whether ministering to the sick or poor in the bosom of your patrons families or the wards of a hospital; in a word—the noble, the skillful, the true physician, worthy of all confidence, worthy of honors, and the fervent benediction of your fellow-men.

"Whatsoever a man soweth, that shall he reap." Let these words ring in clarion tones in your ears as you labor in your special fields of knowledge or action. Let them be as your mentor and stimulator in acquiring your profession, and your "harvest home" will be crowned with a full and noble fruition of your best and highest hopes.

Now, ladies and gentlemen, I have done. The Southern Medical College presents itself to you for kindly words, sympathetic interest, and generous support. Deal with us nobly and justly, and we pledge ourselves, as trustees, faculty and students, to endeavor to make this institution pre-eminently worthy of your confidence, second to none, and the *avant courier* of that period when Atlanta shall become the great medical centre of the South, and its institutions of medicine shall evolve truth from this heaven-born science, whose light and healing will flow out in all directions and be like benisons to suffering humanity from sea to sea of our country's utmost boundaries.

Our beloved Southern land has already a proud name and a famous record wherever civivilization lifts its bright banners to the gaze of the world. Her heroes, statesmen, artists and literateurs have been crowned with the laurel by the noblest and best in Christendom, and that day will surely come when the Sunny South will win the proudest rank in education, whether medical, scholastic or industrial, and take her place as second to none among the nations of the earth.

